# Plant physiological indicators for optimizing conservation outcomes

**DOI:** 10.1093/conphys/coad073

**Published:** 2023-09-12

**Authors:** Leonie Schönbeck, Marc Arteaga, Humera Mirza, Mitchell Coleman, Denise Mitchell, Xinyi Huang, Haile Ortiz, Louis S Santiago

**Affiliations:** Department of Botany & Plant Sciences, University of California, Riverside, CA 92521, USA; Department of Botany & Plant Sciences, University of California, Riverside, CA 92521, USA; Department of Botany & Plant Sciences, University of California, Riverside, CA 92521, USA; Department of Botany & Plant Sciences, University of California, Riverside, CA 92521, USA; Tejon Ranch Conservancy, Frazier Park, CA 93225, USA; Department of Botany & Plant Sciences, University of California, Riverside, CA 92521, USA; Department of Botany & Plant Sciences, University of California, Riverside, CA 92521, USA; Department of Botany & Plant Sciences, University of California, Riverside, CA 92521, USA; Department of Botany & Plant Sciences, University of California, Riverside, CA 92521, USA; Smithsonian Tropical Research Institute, Apartado 0843-03092. Balboa, Ancon, Panama, Republic of Panama

**Keywords:** Drought, photosynthesis, plant eco-physiology, nutrients, temperature, plant hydraulics

## Abstract

Plant species of concern often occupy narrow habitat ranges, making climate change an outsized potential threat to their conservation and restoration. Understanding the physiological status of a species during stress has the potential to elucidate current risk and provide an outlook on population maintenance. However, the physiological status of a plant can be difficult to interpret without a reference point, such as the capacity to tolerate stress before loss of function, or mortality. We address the application of plant physiology to conservation biology by distinguishing between two physiological approaches that together determine plant status in relation to environmental conditions and evaluate the capacity to avoid stress-induced loss of function. Plant physiological status indices, such as instantaneous rates of photosynthetic gas exchange, describe the level of physiological activity in the plant and are indicative of physiological health. When such measurements are combined with a reference point that reflects the maximum value or environmental limits of a parameter, such as the temperature at which photosynthesis begins to decline due to high temperature stress, we can better diagnose the proximity to potentially damaging thresholds. Here, we review a collection of useful plant status and reference point measurements related to photosynthesis, water relations and mineral nutrition, which can contribute to plant conservation physiology. We propose that these measurements can serve as important additional information to more commonly used phenological and morphological parameters, as the proposed parameters will reveal early warning signals before they are visible. We discuss their implications in the context of changing temperature, water and nutrient supply.

## Teaser

Plant physiology can determine the health status of plant species of concern. However, many physiological measurements gain increased context when measured with a reference point that represents the optimal performance and the capacity to withstand stress. Here, we discuss the best practices for plant physiological status and reference point measurements in conservation biology.

## Introduction

Organismal physiology and the capacity to respond to environmental change are critical components of predicting conservation outcomes in species of concern (Madliger *et al.,* 2018). When species are the focus of conservation efforts, it is usually because their populations are reduced enough to raise alarm about their viability (Hayes and Donnelly, 2014; Mcdowell *et al.,* 2016; Keen *et al.,* 2022). Given the increasingly erratic nature of climate change, tenuous population numbers can make it vital to identify species in habitats where environmental anomalies can push them beyond their tolerance limits. It is equally important to initiate conservation efforts once species of concern are identified, and prioritize these efforts against a backdrop of multiple competing needs (Nicotra *et al.,* 2015). Currently, a consensus is emerging that conservation decisions should be based on assessments of the adaptive capacity of species, which incorporate exposure to habitat change and ecological, genetic and physiological sensitivity (Williams *et al.,* 2008). This is based on the reality that with limited funding, conservation priorities must be established. The primary challenge is how to determine the current potential threat and adaptive capacity of contrasting species. This endeavour broadly incorporates aspects of population ecology, genetics and eco-physiological function (Nicotra *et al.,* 2015), as well as the often difficult to forecast whims of natural and anthropogenic forcings. We propose that the physiological status of a species with respect to its reference points provides a robust and dynamically repeatable manner to characterize species of concern for their immediate and long-term risk. These reference points should be related to maximum values or the ability to withstand physiological limits within a community context.

Whereas most conservation efforts are focused on populations (Felton and Smith, 2017), physiological diagnostic measurements are conducted at the individual scale. Individual measurements can quantify health and physiological robustness, but understanding the propensity of a population to respond to environmental change requires measures of multiple individuals to discern a range of environmental resilience. Moreover, resilience to change could be staggered across a population, with some individuals better positioned to respond to change than others (Chardon *et al.,* 2020). Fortunately, between-species trait variation is nearly always greater than within-species trait variation—a fundamental pattern that has enabled trait-based ecology to flourish (Messier *et al.,* 2010).

Comparative physiology of contrasting species within communities can shed light on the competitive potential of species relative to their neighbours, and how that is balanced by their capacity for stress tolerance (Grossiord, 2020). Functional ecology theory informs us that species fall on a spectrum extending from fast-growing, resource-acquisitive species that are prone to risk on one side of the spectrum, to slow-growing, conservative species that are relatively stress tolerant on the other (Reich, 2014; Díaz *et al.,* 2016). Thus, if a species is intermediate for an environmental response trait, it may be buffered by the community. However, if a species stands out in terms of trait vulnerability relative to the community, environmental disruptions could have a disproportionately large effect on that species. This could be a threat to its population viability. When the local processes that structure communities have a stronger effect on community composition than the effect of regional species pools, chance plays a relatively stronger role (Cornell and Harrison, 2014), thus promoting local extinction. Key physiological reference points thus explain where species stand relative to a community and add information to their risk assessment.

**Figure 1:**
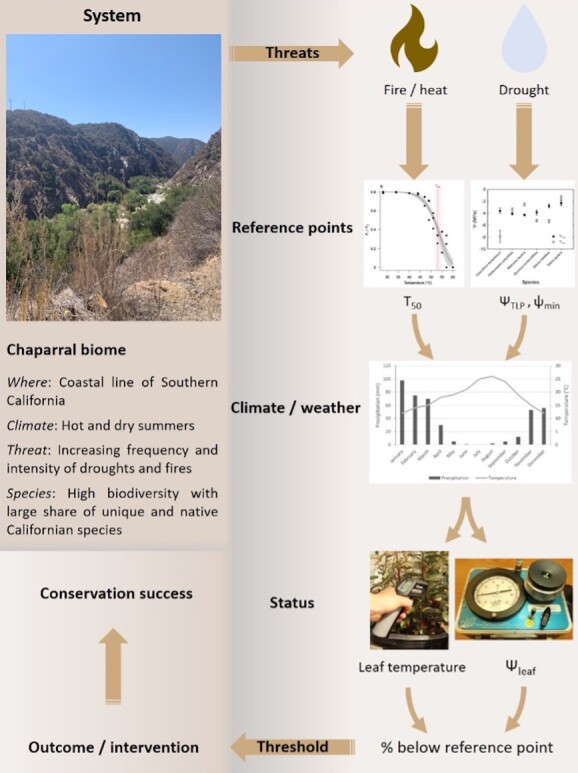
Example of the use of reference points and status indicators in an ecosystem with rare species of interest. The Mediterranean Chaparral system of Southern California is exposed to extreme droughts, heatwaves and fire. The logical reference points for plants growing in this system are thus related to hydraulics and leaf temperature. Adequate selection of reference points can help identify the health status of species in this ecosystem, in turn leading to intervention practices.

Our synthesis addresses the physiological approaches to diagnose the health status and capacity to withstand stress in threatened or managed species. We distinguish between two classes of physiological measurements that, when used together, give us the potential to diagnose the proximity to potentially damaging thresholds. The first is characterization of the instantaneous physiological status of key vital processes such as photosynthetic rate, tissue water status or mineral nutrition. These measures are common in plant eco-physiological studies and are broadly used in agriculture (DaMatta and Ramalho, 2006; Murchie *et al.,* 2009), forestry (Ceulemans and Deraedt, 1999; Colombo and Parker, 1999) and ecology (Koide *et al.,* 1989; Aerts and Chapin, 2000; Maire *et al.,* 2015), with a growing presence in conservation biology (Wikelski and Cooke, 2006). The second represents a physiological reference point such as maximum rates of a physiological process, or the capacity to maintain physiological function in relation to a particular environmental parameter. For example, a measure of leaf water potential indicates plant water status, but without a reference point, it is not immediately clear whether that leaf is undergoing water stress that threatens function. However, when combined with a measure of leaf turgor loss point, the water potential at which the leaf cells lose turgor, or wilt, we can ascertain how close a leaf is to experiencing a stress-induced loss of function (Bartlett *et al.,* 2012b; Kunert *et al.,* 2021; Álvarez-Cansino *et al.,* 2022). In combination with climate and weather data, these assessments contribute to more informed decision making on management (Fig. 1). To better diagnose plant potential to respond to environmental change and contribute to conservation outcomes, we review approaches related to photosynthetic carbon assimilation, plant water relations and mineral nutrition. Our main questions were as follows: (1) How can physiological measurements be structured to capture current, future and comparative performance? (2) Are there particular considerations for diagnosing plant physiological health in a conservation context? (3) How can physiological measurements be incorporated into current and traditional conservation biology approaches, such as analyses of community composition and vegetation monitoring?

### Photosynthesis and productivity

Plant and ecosystem productivity describe the carbon sequestration potential of vegetation, which is the source of carbon income for plant allocation to growth, defence, storage and reproduction (Poorter *et al.,* 2012; Sevanto and Dickman, 2015). Photosynthetic activity is highly sensitive to temperature, vapour pressure deficit (dryness of air; VPD) and soil water availability, with stomatal closure often occurring as an early response to stressful conditions (Martin-StPaul *et al.,* 2017; Agurla *et al.,* 2018). Increasing drought episodes in many locations, combined with rising temperatures and increased VPD, are pushing plant species beyond their climatic history (Loarie *et al.,* 2009; Allen *et al.,* 2010). Overall, while some species can operate within a wide range of water, nutrient availability and temperatures, they often pay the cost of that flexibility through conservative photosynthesis rates (Warren and Adams, 2004). In contrast, other species operate with high temperature sensitivity within narrow thermal ranges (Perez and Feeley, 2020). Therefore, to determine photosynthetic status, physiological reference points and eventual resilience of such species, field measurements are invaluable (Schönbeck *et al.,* 2022).

The rates of leaf photosynthetic carbon assimilation (*A*) and stomatal conductance to water vapour (*g*_s_) under field conditions are usually measured with a portable infrared gas analyser and can be measured at any time to ascertain the current rate of carbon and water exchange with the atmosphere. However, measures of *A* and *g*_s_ that fall within a reasonable range do not signal that a plant is performing well or struggling with carbon assimilation. Therefore, maximum photosynthetic rate (*A*_max_) and stomatal conductance (*g*_s-max_) can provide reference points for interpreting measurements of gas exchange under non-optimal conditions, since it is usually measured under the best possible conditions, which include field conditions on sunny days during mid-morning, before the depression of rates at midday (Mäkelä *et al.,* 1996). In this regard, it is important that measurements are taken consistently at the same leaf age and time of year, as many of these factors change over time (Westoby, 1998). The degree to which *A* and *g*_s_ fall below maximum values is important because stomatal closure is a primary stress response in plants and is the result of a systemic hormonal response driven by abscisic acid (Tardieu and Davies, 1993). When successfully characterized, *A*_max_ and *g*_s_ can serve as reference points and capacity measures for comparison with the status measurements of gas exchange, allowing us to determine how far below optimum values a plant is currently operating. Reference points can differ between ecosystems or even sub-sections of natural areas because they depend on micro-climatic factors and interspecific and intraspecific interactions. A basic knowledge of species composition and micro-climatic variation within natural areas is thus needed to select the optimal conditions for measuring reference points. For example, in a drought-prone area, the most logical timepoint for measuring *A*_max_ would be after a significant rainfall event or at the end of a wet season. Morphological changes over years may also influence the photosynthetic capacity of leaves, as specific leaf area is affected by drought, temperature and CO_2_, with consequences for photosynthetic potential (Li *et al.,* 2013; Luong and Loik, 2022). For this reason, a reconsideration of reference points is useful after one or multiple unusual climatic years.

**Figure 2:**
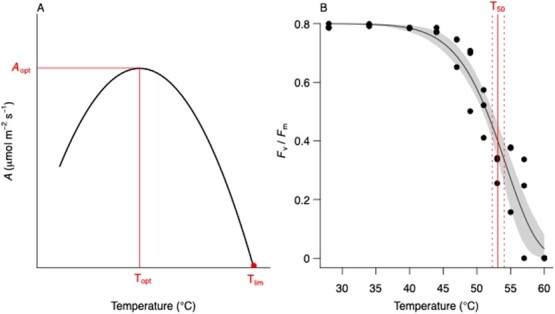
(A) Simplified diagram of photosynthetic CO_2_ assimilation rate as a function of temperature illustrating optimum photosynthetic CO_2_ assimilation rate (*A*_opt_), the optimum temperature at which *A*_opt_ occurs (*T*_opt_), and the temperature at which photosynthesis reaches its limit at the upper CO_2_ compensation point (*T*_lim_). (B) Photosynthetic heat tolerance curve illustrating dark-acclimated leaf chlorophyll fluorescence (*F*_v_/*F*_m_) as a function of increasing leaf temperature to determine the temperature at which 50% of photosynthetic capacity is lost (*T*_50_).

Photosynthetic temperature response curves incorporate gas exchange measures to describe the three physiological reference points of optimum photosynthetic CO_2_ assimilation rate (*A*_opt_), the optimum temperature at which *A*_opt_ occurs (*T*_opt_) and the temperature at which photosynthesis reaches its limit at the upper CO_2_ compensation point (*T*_lim_) (Sage and Kubien, 2007) (Fig. 2A). These curves are typically hump- to parabolic-shaped, where enzymatic activity limits photosynthesis at lower and higher temperatures than optimum values (Fig. 2A) (Medlyn *et al.,* 2002). Above ~45°C, photosynthesis begins to decrease due to chloroplast membrane lipid damage, irreparably damaging the photosystem (Slot and Winter, 2017). These photosynthetic parameters can thus provide an early warning tool for conservationists, as measures of current photosynthetic rate while continuous monitoring of air temperatures give an indication of which plant species are at risk during heatwaves. One recent study also underscores the importance of measuring leaf temperature in concert with air temperature to monitor photosynthetic stress tolerance, since transpiration can cool leaves several degrees below air temperature if water is available (Cook *et al.,* 2021).

*T*_opt_ is usually determined using photosynthetic temperature response curves with gas exchange. In field conditions, *in situ*, such response curves can be accomplished by measuring photosynthesis during the course of heating in the morning (Slot and Winter, 2017). The data can then be fitted according to June *et al.* (2004) and Cunningham and Read (2002) (Table 2)*.* In contrast to photosynthetic temperature response curves, photosynthetic heat tolerance curves use chlorophyll fluorescence to describe the thermal capacity to maintain function under high temperature, with the temperature at which 50% of photosynthetic capacity is lost (*T*_50_) as a comparative reference point (Krause *et al.,* 2010) (Fig. 2B). The *T*_50_ has gained interest in recent years, as more regular and intense heatwaves have exposed plants to temperatures near their thermal tolerance point, something that was rare in the earlier years (Kunert *et al.,* 2021). Whereas photosynthetic temperature response curves require complex measurements of gas exchange, photosynthetic heat tolerance curves can be accomplished using relatively simple chlorophyll fluorescence techniques. One photosynthetic heat tolerance curve protocol that has grown in popularity for its ease involves heating leaf discs to increasing temperatures in a water bath while characterizing the darkened leaf chlorophyll fluorescence (Krause *et al.,* 2010; Perez and Feeley, 2020) (Table 2). Fluorescence measurements also offer ease of interpretation, as values above 0.75 in dark acclimated non-senescent leaf samples generally indicate healthy photosynthesis, and values below 0.75 indicate increasing photo-damage (Table 1). The benefits of fluorescence measurements extend more broadly to the fact that they can be measured remotely in association with vegetation monitoring and applied in non-accessible areas using drones equipped with spectral cameras. Beyond photosynthetic heat tolerance, chlorophyll fluorescence offers a straightforward, powerful and non-destructive tool for screening of plant photosynthetic health status (Makarova *et al.,* 1998; Madliger *et al.,* 2018), as well as early, pre-visual assessment of plant stress, as it detects changes in photoprotection that occurs earlier than leaf browning or shedding due to stress (D'Odorico *et al.,* 2021).

**Table 1:** Status traits that characterize the instantaneous physiological state of plants and corresponding physiological reference points that identify maximum values or the potential for physiological processes to continue with stress imposed by a particular environmental parameter

Status traits	Physiological reference points
*Photosynthesis*	
Instantaneous rate of photosynthetic CO_2_ assimilation (*A*)	Maximum photosynthetic rate under favourable field conditions (*A*_max_) Photosynthetic rate at optimal temperature (*A*_opt_) Temperature of optimum photosynthetic rate (*T*_opt_) Temperature at upper photosynthetic CO_2_ compensation point (*T*_lim_)
Dark acclimated chlorophyll fluorescence	*F*_v_/*F*_m_ > 0.75 signifies healthy photosystem *F*_v_/*F*_m_ < 0.75 signifies photo-damage with greater photo-damage as *F*_v_/*F*_m_ decreases Temperature at 50% loss of photosynthetic capacity (*T*_50_)
*Plant water relations*	
Leaf water potential (Ψ)	Water potential at leaf turgor loss (Ψ_TLP_) Water potential at 50% loss of hydraulic conductivity (Ψ_50_)
RWC	RWC at turgor loss (RWC_TLP_)
Instantaneous stomatal conductance rate (*g*_s_)	Maximum stomatal conductance (*g*_s-max_) Leaf water potential at 50% loss of stomatal conductance (Ψ*g*_s-50_) Leaf water potential at stomatal closure (Ψ*g*_s-close_)
*Mineral nutrition*	
Leaf nutrient concentration	Soil nutrient availability
	Soil pH Soil O_2_ concentration/oxidation–reduction potential

### Plant–water relations, drought resistance, water use and water sources

Climate change-induced plant mortality has become an increasingly important component of conservation physiology due to recent mortality events associated with elevated drought (Allen *et al.,* 2010; Hartmann *et al.,* 2018; Hammond *et al.,* 2022). In addition, where bodies of water have been altered due to anthropogenic activity, changes in water availability, management, or hydroperiod can affect this important resource in species of concern (Mayence *et al.,* 2022). For example, seasonal wetlands such as vernal pools are particularly susceptible to alterations in topography and often provide habitat for rare and endangered species with delicate hydric habitat requirements (King, 1998; Zacharias *et al.,* 2007). Therefore, methods to assess plant water status and capacity to withstand water deficit remain an essential component of the conservation physiologist’s toolbox. Such assessments also provide context as to whether the species of interest is a drought avoider or tolerator, which is indispensable for understanding species positioning in a community (Kooyers, 2015). Monitoring plant water status can inform us with early warning signals of plant drought stress before leaf shedding, phenological adjustments and growth reduction take place.

Plant water status is normally characterized through measurement of plant tissue relative water content (RWC) or water potential (Ψ) (Schulze *et al.,* 1987). Measurements of RWC are simple and can be accomplished with a drying oven and balance on any plant tissue, whereas Ψ requires use of a pressure chamber or psychrometer, limiting the tissue types that are appropriate for measurement (Koide *et al.,* 1989; Rodriguez-Dominguez *et al.,* 2022). Most plant ecophysiologists use Ψ to characterize plant water status because it can be conceptually decomposed into its osmotic and pressure components, which is especially helpful for linking cellular and whole-tissue processes (Bartlett *et al.,* 2012b). More recently, ecophysiologists have taken a fresh look at RWC and suggest that considering plant water pools can deepen our ability to monitor and anticipate mortality risk because it integrates multiple aspects of plant function (Martinez-Vilalta *et al.,* 2019; Sapes and Sala, 2021). However, because of the broad range of tolerable RWC and Ψ values among different species, it is not always immediately obvious how close a particular plant is to dangerous thresholds based on RWC or Ψ measurements alone. Therefore, plant water status measurements are particularly strengthened when accompanied by hydraulic capacity measurements.

**Figure 3:**
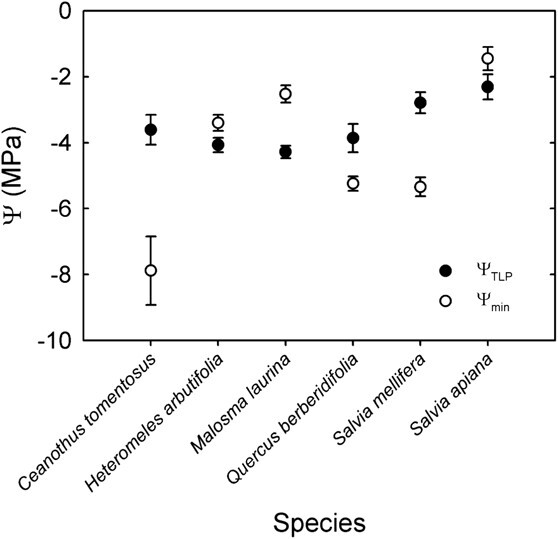
Leaf minimum seasonal water potential (Ψ_min_) and leaf water potential at turgor loss point (Ψ_TLP_) for six species of southern California chaparral shrubs (Schönbeck, unpublished data).

Most plant hydraulic reference point measurements involve characterizing the RWC or Ψ value at which an inflection point in a physiological process occurs. For example, the point at which leaf cells lose turgor, or wilt, can be characterized as the RWC at turgor loss point (RWC_TLP_) or Ψ at turgor loss point (Ψ_TLP_), and have become widely used for characterizing relative potential drought resistance among species (Tyree and Hammel, 1972; Bartlett *et al.,* 2012b). Although it does not necessarily signify a permanent loss of function, it shows ecologically meaningful variation across precipitation gradients and is correlated with drought-induced mortality risk and other key plant hydraulic traits (Baltzer *et al.,* 2008; Bartlett *et al.,* 2016). A comparison of minimum seasonal Ψ with Ψ_TLP_ as a reference point is helpful for placing the most extreme degree of plant water status that a plant experiences into context (Fig. 3). In the example shown in Fig. 3, data for Ψ_TLP_ and minimum seasonal midday leaf Ψ are plotted for six species of chaparral shrubs from southern California to illustrate the increased value of leaf Ψ measurements when combined with a reference point denoting the capacity to withstand stress, in this case the Ψ_TLP_. Here, *Ceanothus tomentosus* Parry, *Quercus berberidifolia* Liebm. and *Salvia mellifera* Greene are shown to have a minimum seasonal Ψ below their leaf Ψ_TLP_, illustrating extreme drought stress with potential to impair leaf function, whereas the other three species maintain Ψ values above their Ψ_TLP_. Measurement of Ψ_TLP_ has increased due to rapid measurement methods, opening the door for a wider cohort of practitioners to characterize drought resistance on species of concern (Bartlett *et al.,* 2012a) (Table 2). However, many plant species show seasonal plasticity in Ψ_TLP_ (Bartlett *et al.,* 2014; Marechaux *et al.,* 2017), so time of measurement is an important consideration. For example, when assessing hydraulic risk in the dry season, it is important to measure dry season Ψ_TLP_ as many species adjust their Ψ_TLP_ in response to drought to lower (more negative) Ψ_TLP_ than wet season values. Other key water-related plant capacity measurements include water-use efficiency (WUE; carbon gained/water lost during photosynthesis), Ψ at 50% loss of hydraulic conductivity (Ψ_50_) and the Ψ at stomatal closure (Ψ_gs-close_) (Tyree and Sperry, 1989; Sack and Holbrook, 2006).

**Table 2:** Measurements, methods and calculation of response curves

Measurement	Instrument	Method	Analysis	Reference
Temperature response	Infrared gas analyser with temperature control	*In situ* measurements during gradual warming early morning to midday	Non-linear exponential equation	Slot and Winter (2017); June *et al.* (2004); Cunningham and Read (2002); Photosynthesis package Stinziano *et al.* (2021); Stinziano & Muir (2022)
Temperature sensitivity	Fluorescence meter such as the miniPAM	Leaf discs exposed to gradually increasing temperature	Weibull/sigmoidal function of F_v_/F*_m_* at corresponding temperature	Archontoulis and Miguez (2015);
Turgor loss point	Osmometer	Leaf discs shock-frozen and punched in osmometer for osmotic potential measurement	Linear regression with pre-determined parameters	Bartlett *et al.* (2012a)
WUE and strategy (C_3_, C_4_, CAM)	Isotopic analysis with isotopic ratio mass spectrometer	Homogenization of dried leaf material and weigh in 1–2 mg in tin capsules before sending off to mass spectrometer.		Mathias and Hudiburg (2022)

Analyses of stable carbon isotopic composition (δ^13^C) can determine whether C_3_, C_4_ or CAM is the major photosynthetic pathway in plants, which strongly structures WUE during photosynthesis. Generally, values of δ^13^C between −33‰ and − 22‰ indicate C_3_ photosynthesis and δ^13^C values between −18‰ and − 8‰ indicate C_4_ photosynthesis, which carries greater WUE and offers a physiological benefit during drought (Ehleringer and Osmond, 1989). Plants with a CAM photosynthetic pathway overlap with C_3_ and C_4_ plants, but can be distinguished by their nocturnal tissue acidification through traditional acid titration (Silvera *et al.,* 2005). At a finer scale within C_3_ plants, δ^13^C scales with photosynthetic WUE, with less negative values indicating greater WUE. However, bulk leaf δ^13^C values, which are commonly used, represent time-integrated measures over the lifetime of a tissue and do not account for short-term stress responses. To account for short term processes, analyses of recent photosynthate δ^13^C in C_3_ plants can reflect daily responses to drought and heatwaves (Snyder *et al.,* 2022). Such physiological reference points thus place information on plant water status into context and provide ancillary information (Table 1).

A key trait that reflects the ultimate capacity for plants with regards to water access is rooting depth (Hasselquist *et al.,* 2010; Pivovaroff *et al.,* 2016a). Belowground traits are inherently difficult to characterize, yet advances in stable isotope techniques now allow estimation of the depth of water uptake. This is accomplished by matching stable isotopic composition of hydrogen and oxygen in water from non-transpiring plant tissues with soil water profiles or alternate water sources that vary in isotopic composition with depth (Allison, 1982; Ehleringer and Dawson, 1992). Downsides to this technique are that it has usually been relegated to woody species and involves destructive sampling, which is often not desired when working with plant species of concern. However, in one study on endangered species along the Amargosa River in California, water was collected by bagging leaves to allow transpired water to condense, and after accounting for evaporative enrichment, the depth of water uptake was estimated non-destructively (Hasselquist and Allen, 2009). Overall, we emphasize that as drought has become an increasingly common component of climate change, such hydraulic measurements have great potential to quantify status and risk of species of concern.

### Mineral nutrition

Soil mineral nutrition differs fundamentally from the physiological status and reference point measurements described above for photosynthesis and water relations. Yet mineral nutrition also interacts with photosynthesis and water transport (Field and Mooney, 1986; Bucci *et al.,* 2006; Pivovaroff *et al.,* 2016b) and may serve as an upper bound for achieving physiological potential at any one site. Plant health in relation to mineral nutrition is based on external sources, thus first understanding the environmental availability of nutrients and how it constrains plant function and physiological capacity is warranted. In this regard, availability of metabolically restrictive elements such as nitrogen and phosphorus can be thought of as determining an ultimate ceiling on physiological potential. This is particularly true for nitrogen, which is energetically costly for plants to store in non-metabolic forms, and is therefore commonly stored as amino acids or proteins, thus necessitating metabolic storage costs (Chapin *et al.,* 1990). In contrast, other elements such as phosphorus and potassium can be stored in ionic forms in vacuoles without disrupting pH or cellular processes (Marschner, 1995; Ostertag, 2010). Thus, luxury consumption, the uptake of mineral elements from soil by plants beyond current physiological needs, can buffer temporal variability in nutrient availability. Specific examples of soil alteration in conservation situations that would necessitate nutrient analyses include restoration in soils affected by pollution, mine tailings, soil waterlogging, plant invasions or when symbiont inoculations such as mycorrhizal fungi or nitrogen-fixing rhizobia have been introduced to facilitate restoration (Neuenkamp *et al.,* 2019; Magnoli and Lau, 2020).

Determining the key soil conditions or elemental concentrations that limit productivity at a site can provide a clear picture of the resource constraints that limit plant growth and the range of physiological rates that can be accomplished at a particular site (McGrath *et al.,* 2014). Whereas nutrient addition experiments that interpret an increase in plant processes such as growth as limitation by that element are normally required to pin down the exact element that limits productivity at a site (Vitousek, 2004), a more accessible method involves measuring the ratio of nitrogen-to-phosphorus concentration (N:P) in leaves, in which values >16 indicate P limitation, values <14 indicate N limitation, and values of 14–16 indicate co-limitation by N and P within a reasonable degree of certainty (Koerselman and Meuleman, 1996; Aerts and Chapin, 2000; Schreeg *et al.,* 2014). In other cases, particular soils such as serpentine, alkali or waterlogged soils may create habitats that are essential for the conservation of unique species that are limited in their range due to habitat requirements (Allen *et al.,* 1997). Such unique soil habitats promote endemism, but can also offer refuge to invasive species with pre-adaptations to local conditions (Batten *et al.,* 2006; Damschen *et al.,* 2012).

Comparison of leaf elemental concentrations with soil nutrient availability of the same element would be a first step in characterizing the overall mineral nutrition situation in a conservation context. Such initial measurements in the context of ancillary data such as site history and conservation status can provide an overall picture of whether intervention is needed. For more detailed mechanistic questions, experiments, often in the greenhouse on potted plants have the potential to isolate specific questions associated with mineral deficiency or imbalance.

### Implementation

**Figure 4:**
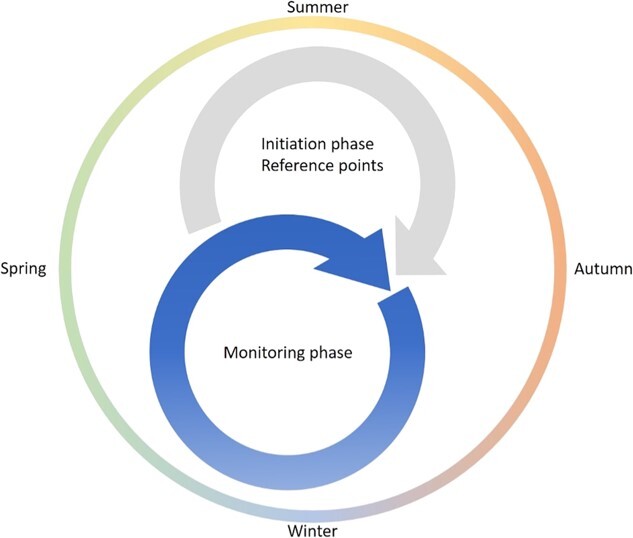
A framework for implementation of the proposed measurements. In year 1, an initiation phase is foreseen where reference points at several timepoints (e.g. spring, summer and autumn, depending on the ecosystem and species studied) are collected. After the first year, monitoring in the seasons of interest on the species of interest can take place with the correct and corresponding reference points at hand, together with the knowledge of the seasonal range of these reference points.

In this review, we propose a tighter connection between plant physiology and conservation practice. Where ecophysiology generally relies on large comparative data sets and replications, investing in higher time-resolution is another way to gain significant information on the health status of species and individuals of interest. For robust and representable measurements, first, a general natural history knowledge of the ecosystem or managed parcel is necessary to apply physiological measurements as an indicator for species status. Site characteristics such as climate, seasonality, edaphic factors and biotic interactions explain why certain species perform better in certain locations. Second, a correct choice of reference point is needed, depending on the ecosystem, species and questions asked. To fully benefit from the physiological approaches outlined in this review, we propose a baseline year for assessment of reference points at specific times per year. Consider an ecosystem with a strong dry season and propensity for drought with measurements beginning at the end of wet and dry season, to assess the extremes in photosynthesis, water status and temperature stress. These measurements can be combined with morphological trait monitoring such as growth, leaf area and greenness. By linking growth to physiological parameters, a better understanding of plant stress and risk can become evident (Manrique-Alba *et al.,* 2018). This baseline information would enable subsequent lower frequency measurements of plant function in following years (Fig. 4). We also acknowledge that some mechanistic questions require measurements under conditions that deviate strongly from ambient would have to be conducted in controlled laboratory conditions, creating important field-lab synergies in the analysis of plant responses to the environment.

## Conclusions

Land managers in many conservation areas are already involved in monitoring, including climate and soil moisture, vegetation surveys, photo surveys, remote sensing and geographic information systems, which are essentially the context for many of the measurements we review (Tomlinson *et al.,* 2021; Merchant *et al.,* 2022). However, most of these measurements are at a scale above what is required to capture the physiological performance of plants. Based on this review of plant physiological approaches for predicting conservation outcomes, we conclude that measuring instantaneous physiological status, coupled with carefully chosen reference points related to key environmental variables specific to the question asked, is a valuable way forward for mechanistically characterizing the health of species of concern. We acknowledge that for many conservationists, physiology represents an approach beyond current instrumentation and training, and thus creative collaborations will be crucial for fully incorporating plant physiological measurements into conservation assessments. We emphasize that the utility of these measurements can be maximized by measuring individuals as part of populations or at a range of sites to understand intra-specific trait variation, and by measuring entire plant communities to determine where a species of concern performs relative to the community. We also note that plant physiological measurements, especially physiological reference points that set an effective standard for future measurements must be taken under the correct conditions to provide sound comparisons. Finally, based on the data presented, we conclude that physiological measurements can best be incorporated into current and traditional conservation biology approaches, such as population viability models, matrix models and analyses of community coexistence by closely matching the scale of study with the question.

## Acknowledgements

We are grateful to the University of California, Riverside; Department of Botany & Plant Sciences; the US Department of Agriculture; and the US National Institute of Food and Agriculture for logistical support. We are also grateful to Jeff Diez, Seth Munson and James Thiede for valuable input on these concepts.

## Author Contributions

L.S.S. and L.S. conceived of the idea based on discussions in our group meeting; D.M., M.C., H.M., M.A., X.H. and HA contributed data to develop the concept; L.S. made the figures in consultation with L.S.S.; and L.S.S. wrote the first draft. All authors edited and wrote parts of the manuscript.

## Conflict of Interest

The authors have no conflicts of interest to declare.

## Funding

We thank the US Fish & Wildlife Service (Q1986003), the US Geological Survey (G19AC00020) and the Swiss National Science Foundation for funding (P500PB_203127).

## Data Availability

No new data were generated or analysed in support of this research.
